# Unmet supportive care needs and associated factors: Evidence from 4195 cancer survivors in Shanghai, China

**DOI:** 10.3389/fonc.2022.1054885

**Published:** 2022-11-30

**Authors:** Minxing Chen, Ruijia Li, Yujie Chen, Gang Ding, Jie Song, Xiaojing Hu, Chunlin Jin

**Affiliations:** ^1^ Shanghai Health Development Research Center, Shanghai Medical Information Center, Shanghai, China; ^2^ School of Public Health, Shanghai University of Traditional Chinese Medicine, Shanghai, China; ^3^ Oncology Department, Shanghai International Medical Center, Shanghai, China

**Keywords:** cancer survivors (MeSH term), unmet supportive care needs, Shanghai, different life stage, patient – centered care

## Abstract

**Background:**

Cancer survivors at different stages of life often have different needs that make it challenging for services to provide satisfactory care. Few studies have considered whether services are truly meeting the needs of cancer patients by exploring and identifying their perspectives on unmet needs.

**Objective:**

The aim of this study was to identify the unmet needs of cancer survivors and to further determine the potential impact of socio-demographic factors.

**Methods:**

A cross-sectional study that included 4195 cancer patients was conducted in Shanghai, China. Using Maslow’s hierarchy of needs theory as a conceptual framework, the questionnaire included five dimensions: information, life and finances, continuing care, emotions, and self-actualization. Correlation analysis and ordered logistic regression analysis was used to explore the relationship between demographic sociological factors and unmet needs for supportive care.

**Results:**

The most common unmet supportive care needs include information needs (2.91 ± 1.32), self-actualization needs (2.69 ± 1.32) and continuing care needs (2.59 ± 1.30). Unmet needs for life and finances were more pronounced among cancer participants in the 45-69 age group. After adjusting for confounders, we found that each 6-month increase in the time since diagnosis was associated with a 0.8% (OR: 0.992, 95% CI: 0.985-0.998) reduction in high need for continuing care and a 0.9% (OR:0.991, 95% CI: 0.983-0.999) reduction in high need for self-actualization, respectively.

**Conclusions:**

Information needs are the most important concern among the diverse unmet needs of cancer survivors. Time since diagnosis is associated with unmet supportive care needs of cancer survivors. The findings highlight the large gap between actual health services and patients’ unmet need for supportive care, which will provide the basis for a patient-centered supportive care system for cancer survivors.

## Introduction

Cancer, as the leading cause of death and an important obstacle to increasing life expectancy in all countries (1), causes a serious burden on the healthcare economy (2). Technological advances such as early cancer screening, targeted therapies, and immunotherapy have contributed to a general increase in the survival period of cancer patients, and the number of cancer survivors has consequently increased (3). However, China ranks first in the world in both the number of new cancer cases and cancer deaths (4, 5), objectively reflecting the poor survival of oncology patients, and there is an urgent need for China to adopt a comprehensive strategy to address the changing cancer burden profile (6).

Patient-centeredness has become the gold standard in the delivery of healthcare worldwide, and effective health policies will help patients to reduce their burden in terms of social life and mental health, including access to health information, financial assistance, social isolation or the burden of caregivers (7, 8). In addition to treatment, comprehensive care for cancer patients should focus on the needs of patients at different levels to facilitate their recovery. Current evidence on the need for health services for cancer survivors remains mixed and incomplete (9). Fiszer et al. reviewed 23 studies on breast cancer patients and found that the information needs and psychological needs of Asian and Western women differed significantly due to their cultural backgrounds (10). Another review suggested that patients with rare cancers have unmet needs throughout their disease trajectory, and their supportive care needs should be addressed individually, depending on the rare cancer subdomain and phase of the disease and from diagnosis onwards (11).

Research studies related to cancer patients in some countries have shown that cancer patients typically have greater unmet needs, which are positively associated with cancer-specific distress (12, 13). The long-term unmet need may substantially reduce patients’ treatment adherence, leading to serious consequences of poorer treatment outcomes, shorter survival, poorer prognosis, and higher risk of recurrence (14–16). A national survey study that included 8,935 Japanese cancer patients showed that younger patients were significantly less satisfied with positive communication with medical staff and with items related to their survivorship in post-treatment care (16). In addition, for patients with rare cancers, delays and extensions in diagnosis were often associated with reduced trust in the professionalism of the patient’s doctor (17).

Previous unmet-need studies conducted in high-income countries such as the US (18), UK (19) and Canada (20) have limited applicability due to the wide variation in healthcare systems and socio-demographic factors. Studies conducted in China (21–24) have been based on qualitative interviews focusing on specific populations, with small sample sizes that do not objectively reflect the comprehensive needs of Chinese cancer survivors and related influencing factors (25). Therefore, we aimed to identify the unmet needs of Chinese cancer survivors, determine the influencing factors, and explore whether there are differences in the needs of survivors at different stages of survival. Based on Shanghai, the largest economic city in China, where 4,195 cancer patients were included, a multidimensional questionnaire was used to analyze the current situation of cancer patients’ needs and to explore the relationship between socio-demographic factors and unmet needs.

## Method

### Study design and data collection

Since 1995, the China Anti-Cancer Association has designated April 15-21 each year as the National Cancer Prevention and Treatment Publicity Week (26). The campaign calls for community-wide attention to the health management of cancer patients and aims to achieve “integrated medicine” from the resources of the medical profession and new technological tools. We surveyed the needs of cancer patients in Shanghai during the 28th National Cancer Awareness Week in 2022 using the online questionnaire. We used quota sampling in the survey, which is a sampling method in which the investigator classifies or stratifies the overall survey sample according to certain markers, determines the sample size for each type (stratum) of units, and draws the sample arbitrarily within the quota. Quota sampling allows for a more balanced distribution of the sample or is more consistent with the overall characteristics (27). Quota sampling was conducted in all areas of Shanghai (16 districts), and 300 questionnaires were distributed by trained research assistants in each district (28). After excluding invalid questionnaires, 4195 questionnaires were included in the final statistical analysis, with a valid response rate of 99.4%.

We recruited adult participants with cancer who had lived in Shanghai for the past three months. Patients were identified and recruited based on their health status at follow-up visits in the past year. The patient is in a stable survivorship phase and is not in urgent need of surgery or radiotherapy (29). The study received ethical approval from the Shanghai Health and Health Development Research Center (Shanghai Institute of Medical Science and Technology Information) under protocol number SHDRC2022005. Due to the restrictions on social distance during the pandemic period, all participants provided informed consent confirmed by electronic signature. Details of the questionnaire can be obtained by contacting the corresponding author.

### Questionnaire

Basic demographic and sociological information on study participants included age, sex education level, marital status, work status, income, and physical activity. The medical information included the location of cancer, time since diagnosis, treatment plan and duration of therapy. The unmet supportive care needs questionnaire for this study was referenced from the Supportive Care Needs Survey-Short Form (SCNS-SF34) (30) and the Short Form for Unmet Needs of Cancer Patients (SF-SUNS) (31). And we simplified and adapted the questionnaire due to language and cultural differences between countries that may affect the measurement of patient-reported outcomes (32, 33).

We conducted Delphi expert consultations to revise our questionnaire in December 2021, January 2022, and March 2022. Experts suggested that we should include cancer survivors’ needs for disease burden and commercial health insurance in the questionnaire scale. Also, it should be ensured that all the contents of the questionnaire are easy to understand for participants with different levels of education. In addition, a pre-survey including 60 participants was conducted to ensure that each question in the questionnaire scale was set to match the Chinese population.

The questionnaire consisted of five need dimensions, information needs (5 entries), living and financial needs (5 entries), continuity of care needs (6 entries), emotional needs (6 entries), and self-actualization needs (2 entries). A five-point Likert scale was used to evaluate these questions, with a maximum score of 5 and a minimum score of 1. A higher score indicates that the patient has a higher level of unmet needs. The total Cronbach’s alpha coefficient of the scale was 0.874, and the coefficients of all dimensions were greater than 0.80. Validity analysis showed that the Kaiser-Meyer-Olkin (KMO) value was 0.978 and was significant (p<0.05), which could be used for factor analysis (34).

### Statistical analyses

Quantitative data from normal distribution were expressed as mean and standard deviation, and differences between groups were compared by double independent samples t-test or one-way ANOVA test. Correlation analyses were performed using Pearson tests of patients’ needs scores in different dimensions. Besides, after adjusting for covariates such as age and sex, we used an ordered logistic regression model to explore the association between time since diagnosis and the unmet need of cancer patients across dimensions. All statistical analyses were performed using R 4.2.1 software with “psych”, “mass”, and “multcomp” packages (35–37). Statistical significance of the tests was reported at p < 0.05.

## Result

### Demographic characteristics

**Table 1** summarized the demographic characteristics of the study participants. The mean age (± SD) of participants was 63.2 ± 7.43 and the age at first diagnosis of cancer was 53.5 ± 8.52. There were more female than male participants (80.4% vs. 19.6%), and more participants with carcinoma *in situ* than metastatic cancer (82.3% vs. 5.5%). The overall unmet supportive care needs score was 61.9 ± 27.9, with significant differences between age groups, for example, participants in the 45-74 age group had higher needs than those in the 18-44 age group (62.4 vs 54.4, p<0.05). The most prevalent cancer diagnosis was breast cancer (39.0%), followed by colorectal cancer (12.8%) and tracheobronchial and lung cancer (10.2%). Details of the cancer diagnoses of the study participants are shown in **Table S1**.

**Table 1:** Basic information and needs scores of study participants.

	Number	%	Total need score (SD)	t/F-value	P-value
Total participants	4195		61.9 (27.9)		
Age, years, mean (SD)	63.2 (7.43)				
Time from initial diagnosis, years, mean (SD)	9.72 (6.42)				
Age at initial diagnosis, years, mean (SD)	53.5 (8.52)				
Age group (years)				2.50	0.04
18-44	65	1.55	54.4 (24.7)		
45-74	3927	93.6	62.4 (28.1)		
≥ 75	203	4.84	58.9 (28.0)		
Sex				0.20	0.58
Males	823	19.6	62.3 (28.6)		
Females	3372	80.4	61.8 (27.8)		
Marital status				0.00	0.76
Married	3636	86.7	61.8 (27.9)		
Single/widowed	559	13.3	61.9 (28.2)		
Education level (years)				0.00	0.99
≤ 9	1933	46.1	61.9 (27.8)		
9-12	1645	39.2	62.0 (28.2)		
≥ 12	617	14.7	61.8 (27.8)		
Working status				0.70	0.54
Employed	162	3.9	59.4 (27.6)		
Retired	3653	87.1	61.9 (28.2)		
Unemployed	380	9.1	62.5 (26.3)		
Average monthly income (RMB)				0.77	
≤ 3000	977	23.3	63.0 (27.8)		
3001 - 6000	2376	56.6	62.2 (28.0)		
6001 - 9000	577	13.8	61.6 (28.1)		
≥ 9000	265	6.3	61.2 (28.2)		
Medical insurance				0.91	0.44
Basic medical insurance	1467	35.0	62.4 (28.1)		
Employee medical insurance	2536	60.5	61.5 (27.9)		
Commercial medical insurance	181	4.3	63.1 (28.2)		
None	11	0.3	71.9 (28.4)		
Physical activity				0.76	0.52
Active	835	19.9	62.5 (28.2)		
Moderately active	744	17.7	61.9 (28.2)		
Mildly active	1751	41.7	61.7 (27.5)		
Sedentary	865	20.6	59.5 (28.4)		
Tumor status				2.50	0.10
Primary tumor	3451	82.3	61.4 (27.9)		
Metastatic tumor	231	5.5	64.1 (28.0)		
Not sure	513	12.2	63.9 (28.6)		
Disease stage				0.70	0.66
Stage I	1629	27.7	62.9 (28.1)		
Stage II	1255	29.9	61.7 (27.8)		
Stage III	752	17.9	61.5 (27.5)		
Stage IV	171	4.1	62.3 (29.5)		
Not sure	855	20.4	61.1 (28.1)		

### Unmet supportive care needs

The results of the descriptive statistics for unmet supportive care needs across the five dimensions are presented in **Table 2**. The most common unmet supportive care needs include information needs (2.91± 1.32), self-actualization needs (2.69 ± 1.32) and continuing care needs (2.59 ± 1.30). In the information needs dimension, 32.7% of patients indicated that it was very important to know about cancer risk factors, with a need score of 3.35, which ranked first in this dimension. In the dimension of the living and financial need, 76.9% and 71.3% of patients indicated a need for detailed information about health insurance reimbursement (Need score: 3.06) and how to receive financial benefits (Need score: 2.89) respectively. Only 29.6% of patients indicated that they needed guidance on sexuality (Need score: 1.71). In the continuity of care dimension, the need for doctors’ appointments was high (Need score: 2.89), with 66.9% and 67.0% of participants indicating the need for care from a community-based family doctor (Need score: 2.63) and reminders for follow-up examinations (Need score: 2.66) respectively.

**Table 2:** Results of descriptive statistics on unmet need for supportive care across five dimensions.

	Frequency (percentage)					Need score (SD)
	No Need	Low Need	Medium Need	Medium-high Need	High Need	
**A. Information needs**						2.91 (1.32)
A1. Oncologist	1185 (28.3)	810 (19.3)	456 (10.9)	707 (16.9)	1037 (24.7)	2.90 (1.57)
A2. Current disease status	1027 (24.5)	892 (21.3)	533 (12.7)	825 (19.7)	918 (21.9)	2.93 (1.50)
A3. Latest treatment	1438 (34.3)	807 (19.2)	529 (12.6)	639 (15.2)	782 (18.6)	2.65 (1.53)
A4. Heredity of the disease	1536 (36.6)	680 (16.2)	422 (10.1)	660 (15.7)	897 (21.4)	2.69 (1.60)
A5. Cancer risk factors	714 (17.0)	742 (17.7)	470 (11.2)	899 (21.4)	1370 (32.7)	3.35 (1.50)
**B. Living and financial needs**						2.49 (1.23)
B1. Time required for treatment	1491 (35.5)	813 (19.4)	478 (11.4)	698 (16.6)	715 (17.0)	2.60 (1.52)
B2. Health insurance reimbursement	971 (23.2)	791 (18.9)	500 (11.9)	896 (21.4)	1037 (24.7)	3.06 (1.52)
B3. Financial benefits	1206 (28.8)	763 (18.2)	492 (11.7)	740 (17.6)	994 (23.7)	2.89 (1.56)
B4. Work situation	2059 (49.1)	711 (17.0)	455 (10.9)	442 (10.5)	528 (12.6)	2.21 (1.45)
B5. Sexual life guidance	2955 (70.4)	399 (9.5)	299 (7.1)	205 (4.9)	337 (8.0)	1.71 (1.27)
**C. Continuing care needs**						2.59 (1.30)
C1. Family doctor care	1390 (33.1)	890 (21.2)	540 (12.9)	627 (15.0)	748 (17.8)	2.63 (1.51)
C2. Follow-up visits	1386 (33.0)	827 (19.7)	525 (12.5)	724 (17.3)	733 (17.5)	2.66 (1.51)
C3. Doctor’s appointment	1239 (29.5)	712 (17.0)	491 (11.7)	767 (18.3)	986 (23.5)	2.89 (1.57)
C4. Rehabilitation care	1545 (36.8)	808 (19.3)	554 (13.2)	633 (15.1)	655 (15.6)	2.53 (1.49)
C5. Psychological support	1958 (46.7)	683 (16.3)	518 (12.4)	447 (10.7)	589 (14.0)	2.29 (1.48)
C6. Privacy protection	1648 (39.3)	712 (17.0)	516 (12.3)	541 (12.9)	778 (18.6)	2.54 (1.55)
**D. Emotional needs**						2.32 (1.23)
D1. Anxiety and depression	2021 (48.2)	762 (18.2)	512 (12.2)	442 (10.5)	458 (10.9)	2.18 (1.41)
D2. Appearance change	1899 (45.3)	843 (20.1)	568 (13.5)	422 (10.1)	463 (11.0)	2.22 (1.39)
D3. Talking about feelings	2038 (48.6)	861 (20.5)	540 (12.9)	372 (8.9)	384 (9.2)	2.09 (1.34)
D4. Communication with patients	974 (23.2)	1217 (29.0)	708 (16.9)	675 (16.1)	621 (14.8)	2.70 (1.37)
D5. Respect	1686 (40.2)	879 (21.0)	613 (14.6)	530 (12.6)	487 (11.6)	2.35 (1.41)
D6. Uncertainty	1535 (36.6)	991 (23.6)	641 (15.3)	498 (11.9)	530 (12.6)	2.40(1.40)
**E. Self-actualization needs**						2.69 (1.32)
E1. Setting new goals	1197 (28.5)	927 (22.1)	674 (16.1)	725 (17.3)	672 (16.0)	2.70 (1.45)
E2. Guidance to help others	1054 (25.1)	1065 (25.4)	805 (19.2)	679 (16.2)	592 (14.1)	2.69 (1.37)

Regarding psychological and emotional well-being, more than half of the participants expressed an urgent need to talk to someone about their feelings and 82.2% of the patients wanted to talk to someone who had similar experiences (Need score: 2.70). In the dimension of self-actualization, 71.5% of patients would like to set new life goals and realize their life aspirations, and 74.9% of patients are willing to share their treatment experience and guide and help their patients.

### Differences in survivor needs between groups

As shown in **Table 3**, age significantly influenced cancer survivors’ need for life and finances, with participants in the 45-74 age group having a higher need for life and finances, and those in the 18-44 age group having a relatively lower need (Need score: 2.51 vs. 2.10, p<0.05). The results showed that participants with metastatic cancer had a higher need for continuity of care than those with *in situ* cancer (Need score: 2.77 vs. 2.57, p<0.05). No significant differences in patients’ needs were found between sex, monthly income, health insurance, and stage of disease. The five dimensions of unmet supportive care needs for information needs, living and financial needs, continuity of care, emotional needs, and self-actualization were correlated with Pearson coefficients of 0.805, 0.812, 0.750, and 0.679, respectively (**Figure S1**).

**Table 3:** Results of univariate analysis of need scores for five dimensions of study participants.

	A. Information needs		B. Living and financial needs		C. Continuing care needs		D. Emotional needs		E. Self-actualization needs	
	Mean Score	P_-value_	Mean Score	P_-value_	Mean Score	P_-value_	Mean Score	P_-value_	Mean Score	P_-value_
Age (years)		0.07		0.03		0.14		0.10		0.14
18-44	2.52		2.10		2.31		2.09		2.48	
45-74	2.92		2.51		2.63		2.37		2.74	
≥ 75	2.80		2.37		2.48		2.18		2.54	
Sex		0.53		0.62		0.25		0.45		0.82
Males	2.91		2.50		2.60		2.36		2.74	
Females	2.90		2.49		2.59		2.32		2.68	
Tumor status		0.06		0.07		0.03		0.43		0.54
Primary tumor	2.88		2.47		2.57		2.31		2.68	
Metastatic tumor	2.99		2.57		2.77		2.37		2.75	
Not sure	3.02		2.59		2.67		2.38		2.74	
Disease stage		0.72		0.57	0.55	0.70		0.45		0.73
Stage I	2.94		2.54		2.63		2.38		2.72	
Stage II	2.89		2.48		2.58		2.32		2.70	
Stage III	2.91		2.48		2.59		2.27		2.65	
Stage IV	2.94		2.50		2.61		2.31		2.77	
Not sure	2.86		2.46		2.55		2.30		2.69	
Marital status		0.85		0.57		0.77		0.68		0.66
Married	2.92		2.47		2.61		2.34		2.73	
Single/widowed	2.90		2.50		2.59		2.33		2.70	
Education level (years)		0.71		0.57		0.87	2.29		2.66	
≤ 9	2.89		2.51		2.59		2.25		2.64	
9-12	2.91		2.47		2.61			0.44		0.53
≥ 12	2.94		2.48		2.58		2.35		2.73	
Working status		0.28		0.14		0.47	2.30		2.67	
Employed	2.75		2.34		2.47		2.36		2.74	
Retired	2.91		2.49		2.60		2.74		3.00	
Unemployed	2.94		2.57		2.58			0.68		0.99
Average monthly income (RMB)		0.45		0.20		0.59	2.30		2.70	
≤ 3000	2.91		2.54		2.62		2.33		2.70	
3001 - 6000	2.90		2.49		2.59			0.88		0.96
6001 - 9000	2.95		2.48		2.59		2.32		2.69	
≥ 9000	2.79		2.36		2.50		2.33		2.70	
Medical insurance		0.61		0.54		0.27	2.30		2.69	
Basic medical insurance	2.91		2.51		2.63			0.86		0.44
Employee medical insurance	2.90		2.48		2.57		2.27		2.74	
Commercial medical insurance	2.96		2.59		2.62		2.33		2.69	
None	3.38		2.71		3.17		2.32		2.77	
Physical activity		0.50		0.54		0.81		0.42		0.38
Active	2.95		2.54		2.61		2.38		2.75	
Moderately active	2.93		2.51		2.60		2.33		2.73	
Mildly active	2.88		2.48		2.60		2.31		2.67	
Sedentary	2.89		2.47		2.56		2.29		2.67	

### Unmet supportive care needs of survivors at different stages

**Figure 1:**
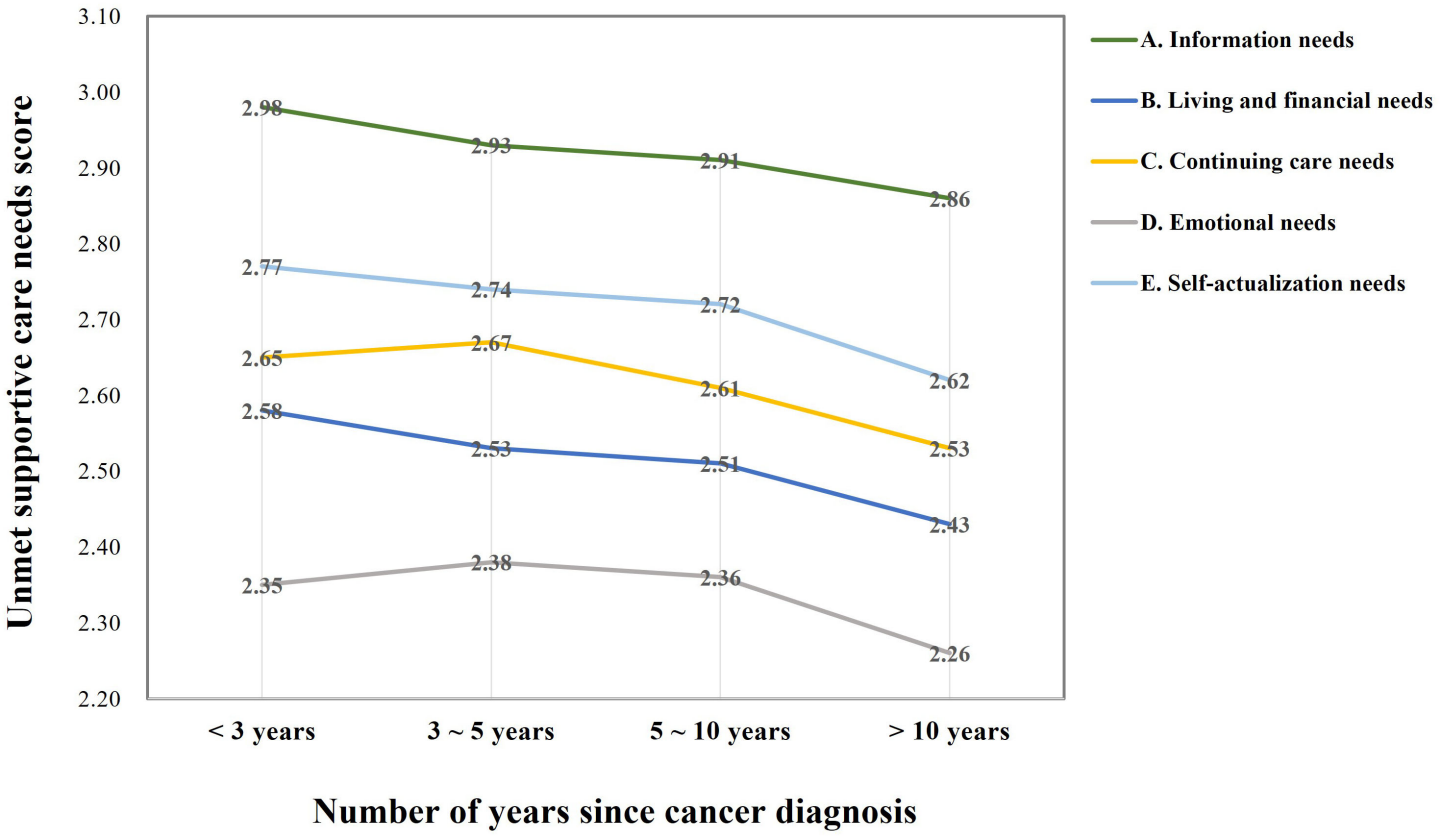
Unmet supportive care needs by time since cancer diagnosis.

The intensity of unmet needs among cancer survivors varies at different stages. Our results showed that the need for continuing care needs and emotional needs dimensions peaks 3-5 years after the cancer diagnosis and gradually decline thereafter. Notably, patients who were first diagnosed less than three years ago (Need score: 2.35) and those diagnosed more than ten years ago (Need score: 2.26) had lower emotional need scores, and those diagnosed 3-5 years ago and 5-10 years ago had higher emotional needs with need scores of 2.38, 2.36, respectively. Other dimensions of cancer survivors’ needs decline over time (**Figure 1**).

**Figure 2:**
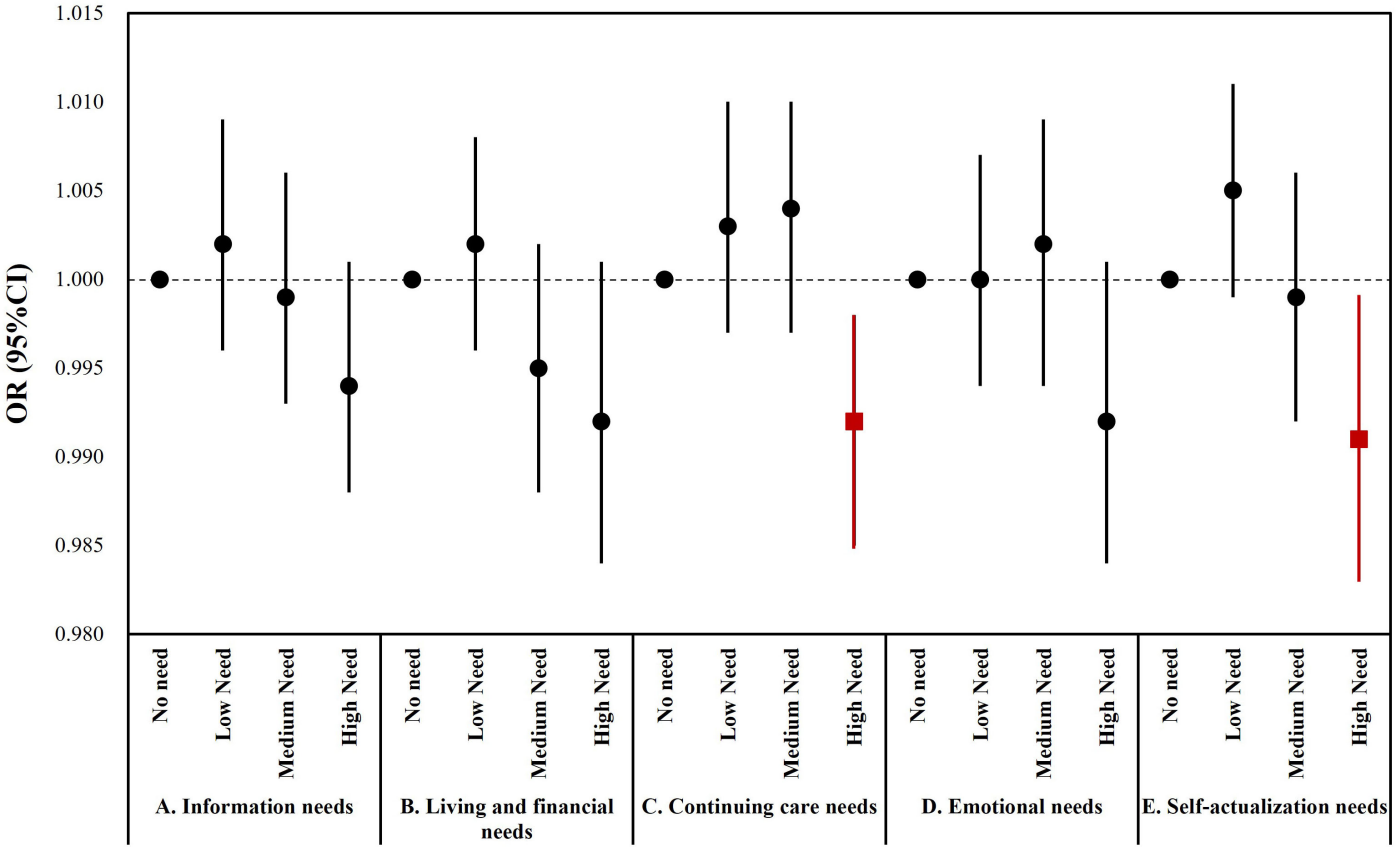
Results of multivariate ordered logistic analysis of the post-diagnosis time and participants’ need level. *Red symbols represent p<0.05. The model was adjusted for age, sex, tumor status, and disease stage.

After adjusting for age, sex, tumor status, and disease stage, we found that each 6-month increase in the time since diagnosis was associated with a 0.8% (OR: 0.992, 95% CI: 0.985-0.998) reduction in high need for continuing care and a 0.9% (OR:0.991, 95% CI: 0.983-0.999) reduction in high need for self-actualization, respectively (**Figure 2**). Although logistic regression analyses of the other need dimensions were not statistically significant, they reflect that cancer patients’ unmet needs may show dynamic changes with time since diagnosis (**Table S2**). The results of the reliability and validity analysis of the questionnaire in this study were presented in **Table S3**, **S4**, respectively.

## Discussion

This study identified the current needs and influencing factors of cancer survivors through a population-based survey study in Shanghai, China. We analyzed the blind spots in the current cancer survivorship management model and explored the differences in the unmet needs of cancer patients at different stages of survivorship. The findings will provide evidence for future exploration to develop a “patient-centered” long-term follow-up management system for cancer survivors.

In our study, the most common unmet supportive care needs include information needs (2.91± 1.32), and 32.7% of patients indicated that the cancer risk factors information was very important. Similar results have been reported in other studies. Icomomou et al. found that Greek cancer patients had a high need for information, particularly about the consequences of chemotherapy, prognosis, how chemotherapy works, how to manage emergencies, everyday preventive measures, and patient psychological support (38). A German study involving 280 participants showed that patients with a high perception of their own control over the disease more often used any source of information available to them and were more often interested in acquiring additional information. Information needs seem to be higher in patients with a high external locus of control and low self-efficacy (39). There is growing agreement that we need to meet the high demand for information from cancer survivors to reduce pessimism and panic due to uncertainty of information (23, 40). However, the quality, availability and visibility of information is difficult to ensure for the various forms of media available. MacLennan et al. proposed a web-based platform to alleviate information silos for cancer survivors in the form of multi-stakeholder engagement, by building a professional community, identifying survivor needs and allowing individuals to actively participate in the design and delivery of supportive care and appropriate information (41). As patients have different perceptions of self-efficacy and control, and information needs vary with these perceptions, future research is expected to take into account and respect these differences when providing structured recovery information guidance to cancer survivors.

Our findings suggested that age factors influence the living and financial needs of cancer patients, with those in the 45-74 age group having significantly higher needs than those in the 18-44 age group and those older than 75 years. Contrary to our research, a retrospective study involving 1129 breast cancer patients did not find differences in financial need across age groups (22). And a study conducted in the Middle East indicated that the score of financial need gradually decreased with increasing age of cancer survivors, but the difference was not statistically significant (42). However, evidence from a review of the quality of survival and unmet need in patients with head and neck cancer, which could support our findings, suggested that older patients have less self-reported unmet needs and lower financial burden compared to younger patients (14). And a cross-sectional international comparative study reported that survivors aged 15–59 years at diagnosis had significantly higher odds of reporting a ‘high/very high’ unmet need for the financial item than survivors aged 60 years and over (43). There are possible explanations for the differences in the level of living and financial needs of cancer survivors across age groups. As younger patients have a lower symptom burden, a better performance status, and a higher quality of life, can return to work more quickly and with relatively less financial stress (44), whereas patients over the age of 45 have a more difficult time returning to work after cancer treatment, they have higher stress levels in terms of forced retirement and have difficulty affording high health insurance (45). Besides, a Canadian study of nasopharyngeal cancer survivors who had completed treatment for more than four years showed that only 62% of patients within working age were still working after diagnosis (≤ 65 years), and nearly a third worked fewer hours than before diagnosis (median decrease of 12 h/week; range, 4-30) (46). The reduction in daily working hours is usually associated with a decline in income, accompanied by ongoing expenses for treatment and rehabilitation, resulting in increased living and financial demands.

Increasing time since diagnosis was associated with a general decline in the need for each dimension among cancer survivors. In our survey, cancer survivors’ need for continuity of care and self-actualization increased up to 5 years after diagnosis, but then declined. Similar to our results, Tzelepis et al. reported that being diagnosed in the last 2 years was significantly associated with an increase in unmet continuity of care need scores (47). An international study conducted in the Asia-Pacific region suggested that higher levels of unmet need were associated with fewer months post-treatment, lower perceived quality of life and higher overall symptom scores (p<0.01) (48). Clinically, the first five years after treatment (transition and extended survival) is a fragile period in which survivors may be caught up in adverse reactions, fatigue, anxiety about the risk of relapse, and life stress (49). One research suggested that cancer survivors have significantly lower needs for supportive care in treatment and at follow-up than at the newly diagnosed stage (22). Some studies in countries with well-developed healthcare systems have also shown high unmet needs among survivors who have just finished treatment, and a decrease among those in recovery (50). Contrary to the above views, a survey of 320 breast cancer survivors in Korea indicated that the level of unmet needs of the advanced cancer patient group was higher than that of the early cancer group in terms of psychological and physical symptoms, social support and hospital services. They attributed this difference to the fact that cancer patients experienced longer and more complex treatments and their side effects at a later stage, and therefore have a greater fear of cancer recurrence and more needs in terms of social and medical resources (51). Cancer type (e.g. *in situ* versus metastatic) and patient mental status also have a greater impact on patients’ unmet needs, and patients with chronic illness or disability tend to be at greater risk and have higher needs later in life (52–54).

One of the strengths of this study is that a quota sample of cancer patients from the whole of Shanghai (16 districts) was included, reducing regionally-induced differences and providing a representative picture of the general situation in the city. As one of the most urbanized cities in China, Shanghai is a model area for healthcare policy with its rich medical resources and level of disease control. Our findings on the unmet needs of cancer patients will provide an evidence-based basis for healthcare decision-making and health service practice. However, there are still limitations to our study. Based on the cross-sectional study design, the inference of causal effects is limited and we cannot further speculate on changes in unmet needs of cancer patients over time. Secondly, adolescent patients were not included in this study and the results may be subject to selection bias. Finally, we used a representative sample of regions rather than a specific cancer dataset. While such a decision strengthens the generalizability of our findings, future efforts should also investigate similar themes using specific cancer datasets to see if our findings are sustained.

## Conclusions

Our findings suggest that the unmet needs of cancer survivors are diverse and complex in China. Information needs were the unmet needs of greatest concern to survivors. An increase in the time since diagnosis was associated with a decrease in patients’ need for continuity of care and self-actualization. We expect that future models of care support for cancer patients should shift from detecting cancer recurrence to improving the quality of life, functional outcomes, experience and survival of cancer survivors, reducing the risk of cancer recurrence and neoplastic disease, improving the management of comorbidities and reducing costs to patients and payers.

## Data availability statement

The datasets presented in this article are not readily available because data sets may violate participants’ privacy. Requests to access the datasets should be directed to chenminxing@shdrc.org.

## Ethics statement

The study was conducted in accordance with the Declaration of Helsinki and approved by the ethics committee of the Shanghai Health Development Research Center (Shanghai Medical Information Center), approval no.: SHDRC2022005. Informed consent was obtained from all subjects involved in the study.

## Author contributions

Writing—original draft preparation: RL. Writing—review and editing: MC. Visualization: YC. Supervision: GD. Investigation: JS and XH. Project administration: CJ. All authors contributed to the article and approved the submitted version.

## Funding

This study was supported by grants from the China Medical Board, “The study of a home-based supportive care system for cancer patients receiving oral chemotherapy” (No. 20-387), and the Shanghai Municipal Health Commission, “Exploration of chronic disease management model for cancer patients in the post-epidemic era”(No. 202240061).

## Acknowledgments

The authors gratefully acknowledge the support of the Shanghai Cancer Rehabilitation Club and all participants of this study.

## Conflict of interest

The authors declare that the research was conducted in the absence of any commercial or financial relationships that could be construed as a potential conflict of interest.

## Publisher’s note

All claims expressed in this article are solely those of the authors and do not necessarily represent those of their affiliated organizations, or those of the publisher, the editors and the reviewers. Any product that may be evaluated in this article, or claim that may be made by its manufacturer, is not guaranteed or endorsed by the publisher.
