# Supplementary material for: Unmet supportive care needs and associated factors: Evidence from 4195 cancer survivors in Shanghai, China
Source: Front Oncol. 2022 Nov 30;12:1054885. doi: 10.3389/fonc.2022.1054885 (PMC9748420; doi:10.3389/fonc.2022.1054885)
Supplement: Supplementary file 1 [file DataSheet_1.docx]

Supplementary Material

**Table S1. Primary and metastatic tumor types of study participants.**

| **Primary cancer** | Metastatic Cancer 1 | Metastatic Cancer 2 | Metastatic Cancer 3 | No. | % |
| --- | --- | --- | --- | --- | --- |
| **Breast cancer** |  |  |  | 1637 | 39.02 |
|  | Thyroid cancer |  |  | 60 | 1.43 |
|  | Tracheal, bronchial and lung cancer |  |  | 50 | 1.19 |
|  | Colorectal Cancer |  |  | 14 | 0.33 |
|  | Ovarian Cancer |  |  | 10 | 0.24 |
|  | Cervical Cancer |  |  | 9 | 0.21 |
|  | Stomach cancer |  |  | 8 | 0.19 |
|  | Pancreatic cancer |  |  | 4 | 0.10 |
|  | Gallbladder/duct cancer |  |  | 3 | 0.07 |
|  | Liver cancer |  |  | 3 | 0.07 |
|  | Ovarian cancer | Gallbladder/duct cancer |  | 3 | 0.07 |
|  | Brain Cancer |  |  | 3 | 0.07 |
|  | Esophageal Cancer |  |  | 3 | 0.07 |
|  | Colorectal Cancer | Ovarian Cancer |  | 2 | 0.05 |
|  | Colorectal cancer | Stomach cancer |  | 2 | 0.05 |
|  | Skin cancer |  |  | 2 | 0.05 |
|  | Bladder Cancer |  |  | 1 | 0.02 |
|  | Liver cancer | Bladder cancer | Brain cancer | 1 | 0.02 |
|  | Liver cancer | Gallbladder/duct cancer |  | 1 | 0.02 |
|  | Cervix cancer | Thyroid cancer |  | 1 | 0.02 |
|  | Colorectal Cancer | Cervical Cancer |  | 1 | 0.02 |
|  | Lymphoma |  |  | 1 | 0.02 |
|  | Prostate Cancer |  |  | 1 | 0.02 |
|  | Kidney cancer |  |  | 1 | 0.02 |
|  | Bladder Cancer |  |  | 1 | 0.02 |
|  | Stomach cancer | Thyroid cancer |  | 1 | 0.02 |
|  | Endometrial Cancer |  |  | 1 | 0.02 |
| **Colorectal cancer** | |  |  | 538 | 12.82 |
|  | Stomach cancer |  |  | 5 | 0.12 |
|  | Thyroid cancer |  |  | 4 | 0.10 |
|  | Liver cancer | Thyroid cancer |  | 3 | 0.07 |
|  | Bladder cancer |  |  | 2 | 0.05 |
|  | Cervical cancer |  |  | 2 | 0.05 |
|  | Prostate cancer | Liver cancer |  | 2 | 0.05 |
|  | Gallbladder/duct cancer |  |  | 1 | 0.02 |
|  | Liver cancer |  |  | 1 | 0.02 |
|  | Liver cancer | Bladder cancer |  | 1 | 0.02 |
|  | Ovarian Cancer |  |  | 1 | 0.02 |
|  | Brain cancer |  |  | 1 | 0.02 |
|  | Prostate cancer |  |  | 1 | 0.02 |
| **Tracheal, bronchial and lung cancer** | |  |  | 429 | 10.23 |
|  | Thyroid cancer |  |  | 19 | 0.45 |
|  | Colorectal cancer |  |  | 15 | 0.36 |
|  | Stomach cancer |  |  | 6 | 0.14 |
|  | Bladder cancer |  |  | 5 | 0.12 |
|  | Cervical cancer |  |  | 5 | 0.12 |
|  | Ovarian cancer |  |  | 4 | 0.10 |
|  | Liver cancer |  |  | 2 | 0.05 |
|  | Brain cancer |  |  | 2 | 0.05 |
|  | Pancreatic cancer |  |  | 2 | 0.05 |
|  | Prostate cancer |  |  | 1 | 0.02 |
| **Stomach cancer** |  |  |  | 290 | 6.91 |
|  | Liver cancer |  |  | 3 | 0.07 |
| **Gastric cancer** | Thyroid cancer |  |  | 3 | 0.07 |
|  | Ovarian cancer |  |  | 2 | 0.05 |
| **Thyroid cancer** |  |  |  | 241 | 5.74 |
|  | Ovarian cancer |  |  | 4 | 0.10 |
|  | Bladder cancer |  |  | 1 | 0.02 |
|  | Nasopharyngeal Cancer |  |  | 1 | 0.02 |
|  | Sweat gland cancer |  |  | 1 | 0.02 |
|  | Brain cancer |  |  | 1 | 0.02 |
|  | Parotid gland cancer |  |  | 1 | 0.02 |
| **Cervical cancer** |  |  |  | 164 | 3.91 |
|  | Bladder cancer |  |  | 1 | 0.02 |
|  | Thyroid cancer |  |  | 2 | 0.05 |
|  | Ovarian Cancer |  |  | 1 | 0.02 |
| **Ovarian cancer** |  |  |  | 110 | 2.62 |
|  | Endometrial cancer |  |  | 5 | 0.12 |
|  | Liver cancer |  |  | 3 | 0.07 |
| **Liver cancer** |  |  |  | 83 | 1.98 |
|  | Thyroid cancer |  |  | 1 | 0.02 |
|  | Pancreatic cancer |  |  | 1 | 0.02 |
| **Prostate cancer** |  |  |  | 37 | 0.88 |
|  | Bladder Cancer |  |  | 1 | 0.02 |
|  | Liver Cancer |  |  | 1 | 0.02 |
| **Bladder Cancer** |  |  |  | 36 | 0.86 |
|  | Gastrointestinal stromal tumor |  |  | 1 | 0.02 |
|  | Brain Cancer |  |  | 1 | 0.02 |
| **Endometrial cancer** |  |  |  | 32 | 0.76 |
| **Kidney cancer** |  |  |  | 31 | 0.74 |
| **Esophageal cancer** |  |  |  | 20 | 0.48 |
| **Nasopharyngeal carcinoma** | |  |  | 20 | 0.48 |
| **Leukemia** |  |  |  | 16 | 0.38 |
| **Lymphoma** |  |  |  | 21 | 0.50 |
| **Pancreatic cancer** |  |  |  | 14 | 0.33 |
|  | Lung cancer |  |  | 1 | 0.02 |
| **Gallbladder/duct cancer** | |  |  | 10 | 0.24 |
| **Brain cancer** |  |  |  | 6 | 0.14 |
|  | Ovarian Cancer |  |  | 1 | 0.02 |
| **Oral Cancer** |  |  |  | 4 | 0.10 |
| **Skin cancer** |  |  |  | 4 | 0.10 |
| **Fallopian tube cancer** |  |  |  | 2 | 0.05 |
| **Thymus cancer** |  |  |  | 2 | 0.05 |
| **Penile cancer** |  |  |  | 1 | 0.02 |
| **Other** |  |  |  | 138 | 3.29 |
| **Total** |  |  |  | 4195 | 100.00 |

**Table S2. Results of multivariate ordinal logistic analysis of time since diagnosis and participant needs (per 6 months increase)***

| Need dimension |  | OR | 95%CI | P-value |
| --- | --- | --- | --- | --- |
| A. Information needs | No need | Reference |  |  |
|  | Low Need | 1.002 | (0.996,1.009) | 0.524 |
|  | Medium Need | 0.999 | (0.993,1.006) | 0.863 |
|  | High Need | 0.994 | (0.988,1.001) | 0.107 |
| B. Living and financial needs | No need | Reference |  |  |
|  | Low Need | 1.002 | (0.996,1.008) | 0.533 |
|  | Medium Need | 0.995 | (0.988,1.002) | 0.139 |
|  | High Need | 0.992 | (0.984,1.001) | 0.068 |
| C. Continuing care needs | No need | Reference |  |  |
|  | Low Need | 1.003 | (0.997,1.010) | 0.340 |
|  | Medium Need | 1.004 | (0.997,1.010) | 0.270 |
|  | High Need | 0.992 | (0.985,0.998) | **0.038** |
| D. Emotional needs | No need | Reference |  |  |
|  | Low Need | 1.000 | (0.994,1.007) | 0.965 |
|  | Medium Need | 1.002 | (0.994,1.009) | 0.681 |
|  | High Need | 0.992 | (0.984,1.001) | 0.072 |
| E. Self-actualization needs | No need | Reference |  |  |
|  | Low Need | 1.005 | (0.999,1.011) | 0.125 |
|  | Medium Need | 0.999 | (0.992,1.006) | 0.793 |
|  | High Need | 0.991 | (0.983,0.999) | **0.020** |

* The model was adjusted for age, sex, tumor status, and disease stage.

**Table S3. Results of the reliability analysis**

|  | Cronbach's Alpha | Cronbach's Alpha Based on Standardized Items | N of Items |
| --- | --- | --- | --- |
| Total | 0.874 | 0.874 | 24 |
| A. Information needs | 0.910 | 0.910 | 5 |
| B. Living and financial needs | 0.893 | 0.891 | 5 |
| C. Continuing care needs | 0.927 | 0.927 | 6 |
| D. Emotional needs | 0.847 | 0.847 | 6 |
| E. Self-actualization needs | 0.864 | 0.864 | 2 |

**Table S4. Results of the validity analysis**

| KMO value |  | 0.978 |
| --- | --- | --- |
| Bartlett's sphericity test | Approximate cardinality | 97484.972 |
|  | Degree of freedom | 276 |
|  | Significance | 0.000 |

Abbreviation: KMO, Kaiser-Meyer-Olkin;

*
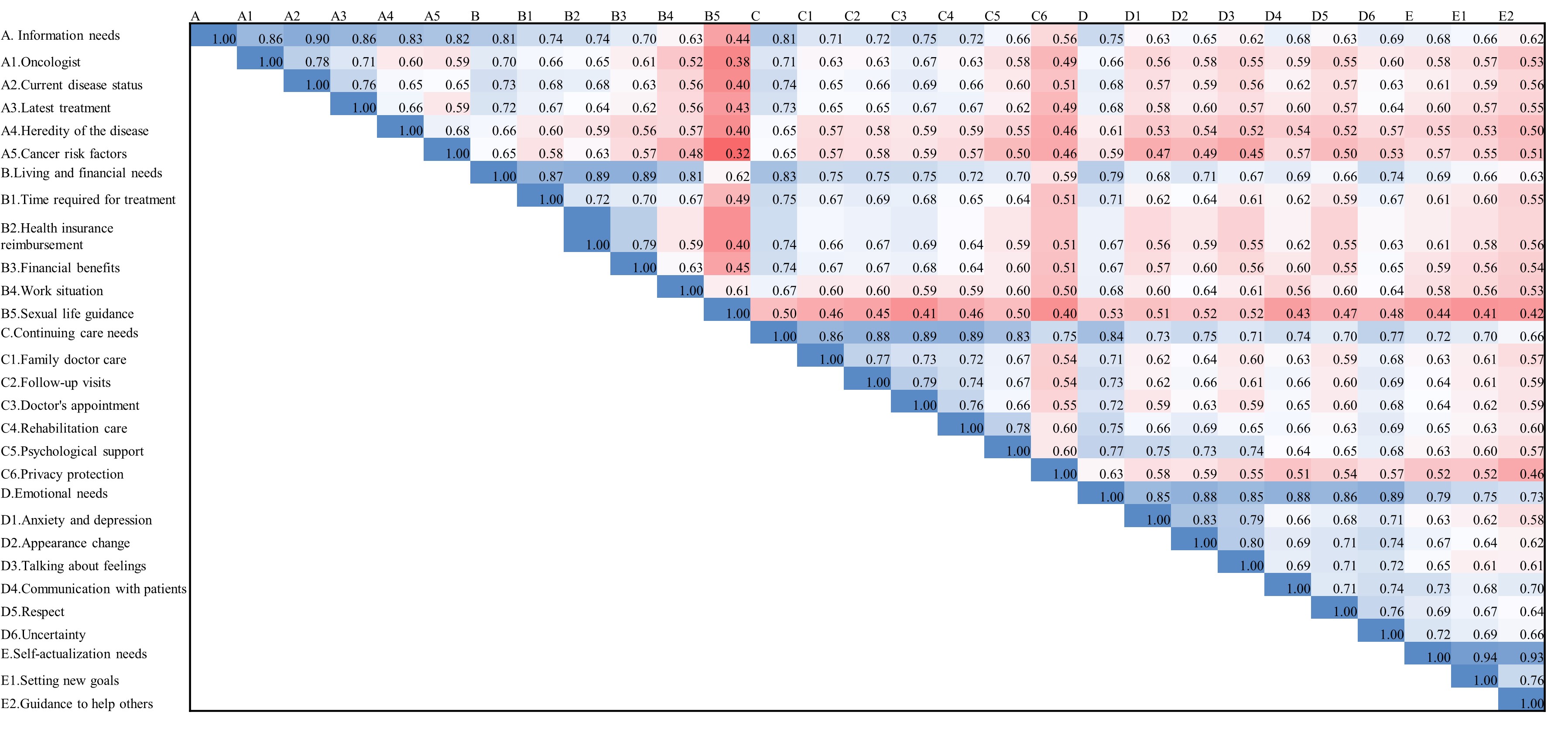
*

**Figure S1. Results of the Spearman Correlation Analysis of Participant Unmet Supportive Care Need. *p<0.05.**
